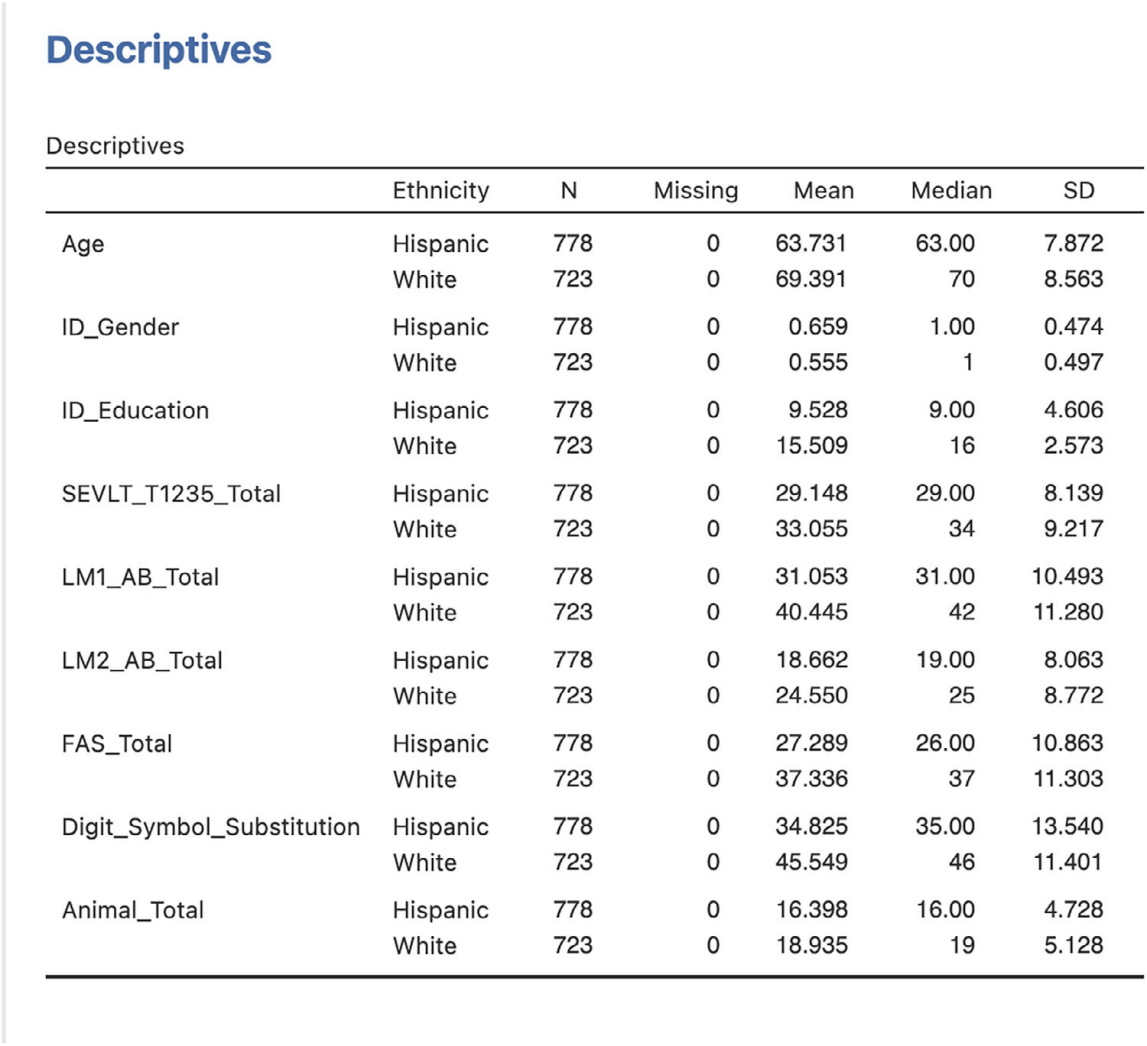# Examining Genetic Differences in Alzheimer’s Disease in Mexican American Older Adults

**DOI:** 10.1002/alz70861_108221

**Published:** 2025-12-23

**Authors:** Kimberly Espejo, Iris J. Broce, Marcella Barneclo, Joey A Contreras, Judy Pa

**Affiliations:** ^1^ Alzheimer’s Disease Cooperative Study (ADCS), University of California, San Diego, La Jolla, CA USA; ^2^ Department of Neurosciences, University of California, San Diego, La Jolla, CA USA; ^3^ Alzheimer's Disease Cooperative Study (ADCS), University of California, San Diego, La Jolla, CA USA

## Abstract

**Background:**

Hispanic/Latino Americans are 1.5 times more likely to develop Alzheimer’s Disease (AD) than non‐Hispanic Whites (NHWs), yet they remain understudied in AD research, limiting the generalizability of findings and the efficacy of treatments. The *APOE4* allele exemplifies these group differences. While *APOE4* is associated with increased AD risk, its frequency and cognitive effects vary by subgroup population. Other genetic risk factors for AD may also differ by ethnicity. A previous study using the Healthy Aging Brain Study ‐ Health Disparities (HABS‐HD) cohort found significant genotype frequency differences between Mexican Americans (MA) and NHWs. The present study examined the relationship between 6 of the top ten AD risk SNPs and cognition and how this relationship differs by ethnicity.

**Method:**

Using the HABS‐HD cohort, this study investigated whether AD‐associated risk alleles in *BIN1* (rs6733839), *CD2AP* (rs9473117), *PTK2B* (rs73223431), *FERMT2* (rs17125924), *NYAP1* (rs12539172), and *CR1* (rs4844610), were differentially related to cognitive performance across MAs and NHWs. Genotype data were generated using GSAv3 and imputed using the TOPMed. Linear regression models tested the interaction between SNP and ethnicity on 6 cognitive outcomes: Spanish‐English Verbal Learning Test Trials 1‐5 (SEVLT1‐5), Logical Memory I and II, F‐A‐S test, Animal Fluency, Digit Symbol Substitution Test. Thirty‐six models were tested and controlled for age, gender, education, and APOE4 status. Models with *p* <0.2 for interactions were followed by post‐hoc comparisons. Significance was set at *p* <0.05.

**Result:**

The risk allele in *BIN1* was linked to poorer performance on Logical Memory I and II among NHWs (*p* = 0.016; *p* = 0.037), but not in MAs. Similarly, the risk allele in *CD2AP* was linked to lower Logical Memory I scores in NHWs (*p* = 0.029), and not in MAs (*p* = 0.9840). The risk allele in *PTK2B* was associated with higher SEVLT1‐5 scores among MAs (*p* = 0.049), with no effect on NHWs (*p* = 0.8391).

**Conclusion:**

These findings suggest that the cognitive effects of AD risk alleles may differ by ethnicity, highlighting the importance of inclusive genetic research to improve risk profiling and care.